# The National Women's Health Study: assembly and description of a population-based reproductive cohort

**DOI:** 10.1186/1471-2458-4-35

**Published:** 2004-08-07

**Authors:** Noreen Maconochie, Pat Doyle, Susan Prior

**Affiliations:** 1 Department of Epidemiology and Population Health, London School of Hygiene and Tropical Medicine, Keppel Street, London WC1E 7HT, UK

## Abstract

**Background:**

Miscarriage is a common event but is remarkably difficult to measure in epidemiological studies. Few large-scale population-based studies have been conducted in the UK.

**Methods:**

This was a population-based two-stage postal survey of reproductive histories of adult women living in the United Kingdom in 2001, sampled from the electronic electoral roll. In Stage 1 a short "screening" questionnaire was sent to over 60,000 randomly selected women in order to identify those aged 55 and under who had ever been pregnant or ever attempted to achieve a pregnancy, from whom a brief reproductive history was requested. Stage 2 involved a more lengthy questionnaire requesting detailed information on every pregnancy (and fertility problems), and questions relating to socio-demographic, behavioural and other factors for the most recent pregnancy in order to examine risk factors for miscarriage. Data on stillbirth, multiple birth and maternal age are compared to national data in order to assess response bias.

**Results:**

The response rate was 49% for Stage 1 and 73% for the more targeted Stage 2. A total of 26,050 questionnaires were returned in Stage 1. Of the 17,748 women who were eligible on the grounds of age, 27% reported that they had never been pregnant and had never attempted to conceive a child. The remaining 13,035 women reported a total of 30,661 pregnancies. Comparison of key reproductive indicators (stillbirth and multiple birth rates and maternal age at first birth) with national statistics showed that the data look remarkably similar to the general population.

**Conclusions:**

This study has enabled the assembly of a large population-based dataset of women's reproductive histories which appears unbiased compared to the general UK population and which will enable investigation of hard-to-measure outcomes such as miscarriage and infertility.

## Background

Despite improvements in obstetric care in the UK over the past fifty years, it is estimated that around one in five pregnancies will end in miscarriage (fetal death before 24 weeks) [1,2]. The personal and public health impact of pregnancy loss is a neglected area in medical research and strategies of prevention remain outside mainstream medical services.

Although many large-scale population-based studies of miscarriage risk have been conducted elsewhere [3-10], relatively few such studies have been conducted in the UK, and most of these have been occupational [11-14]. There are no registers of miscarriage or routine data collection systems which would allow linkage of miscarriages to individual women in the UK . There are thus no national prevalence estimates which can be used as reference for UK-based clinical or epidemiological studies. In addition, although there is now greater knowledge of how the risk of miscarriage changes with maternal age and previous history of miscarriage [6], the influence and interaction of biological, behavioural and social risk factors are less well-understood. The lack of reliable information on risk factors, and the confusion surrounding ad hoc reports of spurious associations, makes research in this area of great importance.

Studies of miscarriage have tended to be clinical-based, and are thus subject to selection bias. For example, gestations are later among miscarriages reaching hospital-based clinics. Many miscarriages are managed at home, and some are not reported to a clinician. Not only is miscarriage hard to measure, and different clinical sources rarely see the full range of cases, but reported risks of miscarriage tend to be pregnancy-rather than woman-based: estimates of risk tend to relate to the proportion of pregnancies ending in miscarriage, and there are very few studies examining the risk of experiencing one, two or more miscarriages, or the chances of conceiving following a miscarriage [15]. Large prospective cohort studies are theoretically the ideal design, but take time and are prohibitively expensive [2]. An alternative and practical approach is a survey asking the women themselves for their full reproductive history, including all fetal losses at all gestations.

An increasing number of couples are also seeking help for problems achieving a pregnancy. Although it is estimated that up to 15% couples experience such problems [16], few population-based prevalence studies have been conducted in the UK, particularly where fertility problems have been treated solely by the general practitioner using ovarian stimulation.

We now report on a large UK population-based survey of reproductive health, the National Women's Health Study. The study design was developed from several other large epidemiological surveys of reproductive outcome which showed that a postal method could be used to obtain full reproductive histories from large study populations [13,14,17,18]. The aim of the study was to obtain population-based prevalence estimates relating to miscarriage and infertility, and to obtain good quality data on potential risk factors for miscarriage to be used when advising and counselling women who have suffered miscarriage and those who wish to reduce their risk of future pregnancy loss. The design of the study, together with response rates and description of the study population, is presented in this report. Further reports on risk factors for miscarriage, plus population-based estimates of miscarriage and of pregnancies conceived using assisted reproduction techniques will follow.

## Methods

### Sample selection

This was a population-based cross-sectional postal survey of reproductive histories of adult women living in the United Kingdom in 2001, designed to enable the construction of a retrospective population-based reproductive cohort and a case-control study of risk factors for miscarriage. A sample of women was randomly selected from electronic electoral registers for England, Wales, Scotland and Northern Ireland held by the company *Eurodirect *[19]. All UK citizens aged 18 and over are eligible to vote; registration is voluntary, but in 2001 around 98% of the entire resident population were on the electoral register [20], the remainder being largely non-UK citizens and iterant population. At the time of survey there was no opt-out clause for those who did not wish to be on an electronic version of the electoral register, so the sampling frame contained all UK residents eligible (and registered) to vote.

In order to reduce possible biases associated with memory, we aimed for a sample aged 55 years and under at survey. Date of birth is not, however, routinely recorded on the electoral register. To avoid unnecessary mailing and expense, we therefore made use of a probabilistic process offered by *Eurodirect *based on forename, whereby the sampling frame was restricted to women thought likely to be aged 55 and under on the basis of their name. This process was based on empirical data relating to birth certificates going back to the beginning of the 20th century, from which it could be calculated that, for example, those named "Elsie" are likely to be aged over 55, and those named "Kylie" under 55 years. Predictions are further refined by examination of combinations of names within a household (a "Jane" married to or living with an Alfred likely to be older than a "Jane" married to or living with a "Darren") and length of residency (e.g. someone registered to vote at the same address for 12 years has to be over 30). We requested a random sample of 61,000 women likely to be aged 55 and under (sample size calculations based on achieving at least 80% power for key risk factors in the case-control analysis, and cost). After removing those known to be under age 18 at study (those turning 18 in the year of registration are allowed to register early, giving date of birth), the final sample consisted of 60,814 women.

The study received approval from the Trent Multi-Centre Research Ethics Committee and the Ethics Committee of the London School of Hygiene & Tropical Medicine.

### Postal survey

The postal survey had two stages. Stage one consisted of a single-page "screening" questionnaire which asked for details of all pregnancies experienced by study participants, as well as periods of infertility and infertility treatment. This form was sent to the whole sample and included "opt-out" boxes to be ticked if the recipient had never been pregnant and had never attempted to have children, and/or was over age 55, and/or did not wish to take part. The second stage of the study consisted of a longer postal questionnaire which was sent to all those responding to Stage 1 who had ever been pregnant or who reported ever attempting to conceive and who agreed to be re-contacted. Excluded from this second stage were women who had had one or more termination for non-medical reasons (i.e. for reasons other than that a defect had been identified in the fetus or that continuing the pregnancy would put the mother at risk) and no other pregnancies. The Stage 2 questionnaire requested more general detail about the women (including height, age at menarche, educational level, marital status and details of infertility problems, treatment and diagnosis, if appropriate); detailed information on all pregnancies (including whether the pregnancy was the planned, the result of infertility treatment, father's date of birth and whether father had remained the same); plus socio-demographic and behavioural details relating to the most recent pregnancy. These details included questions relating to weight at start of pregnancy, nausea, smoking, coffee and alcohol consumption, diet, vitamin intake, ill health, air travel, sexual intercourse, occupation and stress levels. The most recent pregnancy was selected to minimise biases related to recall, and since it could be at the start, middle or end of the reproductive careers of these women whose ages at survey ranged from 18 to 55 years potential biases relating to ending reproductive careers on a "success" were not expected to be large. For those whose most recent pregnancy had ended in miscarriage (defined as fetal death at <24 weeks gestation), brief information relating to clinical management of miscarriage and the advice given was also requested. Permission to access clinical notes relating to outcomes reported in the questionnaire, and to contact the women for further study if needed, was also requested. In order to increase the number of cases for the case-control analysis of risk factors for miscarriage, women who had had a miscarriage recently (since 1995) but whose last pregnancy was not a miscarriage were sent a third questionnaire. This was a shortened version of the Stage 2 questionnaire, containing only those questions relating to biological, socio-demographic and behavioural details of the most recent pregnancy, but now requesting these details in relation to the most recent miscarriage. Such women then had two pregnancies in case-control analyses and standard errors were computed using a robust method based on the "sandwich estimate" to account for this statistically.

A free telephone helpline was run throughout the study, to answer queries and refer on to other organizations for professional help, if appropriate, and this was well used.

### Statistical methods

All analyses in this paper were performed using Stata statistical software [21]. To investigate possible selection bias we compared stillbirth and multiple delivery rates with rates in the general population. For this we obtained annual registered stillbirth risks and registered multiple delivery rates by maternal age for England and Wales, 1980–2001 [22] (data for 2002 was estimated from that for 2001). Standardised registered stillbirth ratios (SRSR) and standardised multiple delivery rates (SMDR) were then calculated using logistic regression analysis (offsetting the log odds of the population risk) [23]. The unit of analysis for stillbirths was a registered birth. A registered livebirth is defined as a baby born alive at any gestation, registered stillbirth being defined as a fetal death at 28 weeks or more gestation until the end of 1992, and at 24 weeks or more gestation from 1993 onwards. Where gestational age was not available from Stage 2 data, a pregnancy was considered to be a stillbirth if it was so described. Forty-one (40%) of the total 102 stillbirths in the analysis fell into this category. For multiple delivery, the unit of analysis was a pregnancy containing at least one livebirth or registered stillbirth (as described above). For the purposes of the analyses presented in this paper (comparisons with the general population), a pregnancy was only considered multiple if it contained two or more babies who were liveborn or (registered) stillborn in order to be consistent with the definitions used in the national data. Thus, for example, a twin pregnancy occurring before 1993 and resulting in a livebirth and a fetal death at less than 28 weeks was considered to be a singleton pregnancy in this analysis. Average maternal age at first birth, if live, was also compared with that in the general population. Annual average maternal age at first (registered) birth, if live, was obtained with denominators for England and Wales, 1980–2001 [22] and re-calculated for 5-year periods. This national data was available for births within marriage only. Marital status of mother at time of birth was known only for the most recent pregnancy (or most recent miscarriage since 1995) in this dataset. For the NWHS average maternal age was therefore calculated for all first registered births, if live. No formal statistical comparisons of maternal age were made, partly because the numbers were so large that slight, non-meaningful, nuances in the data would give a statististically significant result, and render the comparison meaningless, and partly because the average ages in the general population, though comparable, were expected to be similar but slightly older in the general population data owing to the fact that the data related to births within marriage only. Births where the date of birth or maternal age were not known were excluded from all comparisons with population data.

## Results

### Stage 1

The response to the first stage of the study is summarised in Table 1. 29,721 (49%) of all the questionnaires were returned to us, though for 3,591 (6%) this was to say that the addressee had moved, and for 70 (0.1%) that the woman had died. A total of 26,050 questionnaires were returned by the addressee, a response rate of 46% assuming that all questionnaires not returned undelivered had reached the correct recipient. Of these, 11% (5% overall) did not wish to participate in the study, and a further 21% were aged over 55 (n = 5,499) or were otherwise ineligible (n = 65). 27% of the 17,748 women who were eligible on the grounds of age, reported that they had never been pregnant and had never attempted to conceive a child, the remaining 13,035 women reporting their full reproductive history.

**Table 1:** The National Women's Health Survey – response rates

**STAGE 1**	No.	Crude %	Adjusted^1 ^%
*TOTAL QUESTIONNAIRES POSTED*	*60,814*	*100%*	-
Returned undelivered^2^	3,661	6%	-
Responded	26,050	43%	46%
Did not wish to participate	2,738	5%	5%
Aged >55 years or otherwise ineligible^3^	5,564	9%	10%
Aged < = 55 years but never attempted to have children	4,713	8%	8%
Aged < = 55, ever attempted to have children	13,035	21%	23%
*Among whom,*			
*- Never pregnant*	*340*	*3%*	-
*- Ever pregnant*	*12,695*	*97%*	-

**STAGE 2**			

*TOTAL QUESTIONNAIRES POSTED*	*10,828*	*100%*	-
Returned undelivered	16	0.2%	-
Responded	7,882	73%	73%
No longer wished to participate	180	2%	2%
Completed questionnaire	7,702	71%	71%
*Among whom,*			
*- Attempted pregnancy, never pregnant*	*194*	*3%*	
*- Ever pregnant*^4^	*7,508*	*97%*	

^1 ^Adjusted for undelivered mail ^2^ Includes 70 women who died before the study start ^3^ Under 18 at study start (6^th ^November 2001); male; foreign national; or too ill to participate ^4^ 344 women who had had a miscarriage since 1995, but whose last pregnancy was not a miscarriage, were sent a second stage 2 questionnaire and were asked to supply details in relation to their most recent miscarriage. 285 (83%) of the women responded to this third questionnaire.

12,695 women aged under 55 at survey had been pregnant at least once. These 12,695 women, whose average age at survey was 40.5 years, had started their reproductive careers from 1963 to 2002, 75% having their first pregnancy in 1980 or later (Table 2). 486 women had conceived their first pregnancy less than 40 weeks before the study commenced, 126 of whom were pregnant when they filled in the questionnaire. Overall these 12,695 women reported a total of 30,661 pregnancies, 80% of which occurred in 1980 or later. Outcome of these pregnancies is described in Table 2.

**Table 2:** NWHS Stages 1 and 2 – description of women reporting one or more pregnancy, and of the pregnancies they reported

	**STAGE 1**		**STAGE 2**	
	**n**	**(%)**	**n**	**(%)**
**TOTAL NO. WOMEN IN ANALYSIS**	**12,695**	**(100)**	**7508**	**(100)**
**Age at survey (years)**				
<30	1247	(9.8)	685	(10.6)
30–34	2007	(15.8)	1284	(20.6)
35–39	2618	(20.6)	1629	(28.6)
> = 40	6678	(52.6)	3910	(39.3)
Not known	145	(1.1)	-	
*Mean age (SD)*^1^	*40.5 (8.45)*		*40.4 (8.24)*	
**Year of first pregnancy**				
<1980	3201	(25.2)	1798	(24.0)
1980–84	1902	(15.0)	1131	(15.1)
1985–89	2091	(16.5)	1259	(16.8)
1990–94	2158	(17.0)	1356	(18.1)
1995–99	2079	(16.4)	1406	(18.7)
2000–02	788^2^	(6.2)	558^3^	(7.4)
Not known	476	(3.8)	-	
**Total number of pregnancies reported per woman**				
1	2607	(20.5)	1403	(18.7)
2	5077	(40.0)	3162	(42.1)
3	2962	(23.3)	1749	(23.3)
4	1573	(12.4)	818	(10.9)
5	285	(2.2)	229	(3.1)
> = 6	191	(1.5)	147	(1.9)
*Median (range)*	*2 (1 – 18)*		*2 (1 – 18)*	
**Pregnancy history**				
No dates given for any pregnancies	436	(3.4)	-	
All pregnancies occurred before 1980	1495	(11.8)	853	(11.4)
Pregnancies before and after 1980	1707	(13.5)	945	(12.6)
Pregnancy history commenced 1980 onwards	9057	(71.3)	5710	(76.1)
*All pregnancies conceived after 31/03/2000*	*486*	*(3.8)*	*329*	(4.4)
**TOTAL REPORTED PREGNANCIES**	**30661**	**(100)**	**18391**	**(100)**
**Outcome of pregnancy**				
Livebirth, surviving >7 days	24081	(78.9)	14782	(80.4)
Livebirth, early neonatal death	95	(0.3)	56	(0.3)
Stillbirth	188	(0.6)	110	(0.6)
Miscarriage^4^	3512	(11.5)	2326	(12.7)
Ectopic	226	(0.7)	102	(0.6)
Termination for medical reasons^5^	312	(1.0)	89	(0.5)
Termination for non-medical reasons^6^	1424	(4.6)	562	(3.1)
Molar pregnancy	47	(0.2)	26	(0.1)
Ongoing (current) pregnancy	482	(1.6)	338	(1.8)
Not known	294	(1.0)	-	
**Year of pregnancy end**				
<1980	6093	(19.9)	3486	(18.0)
1980–84	4503	(14.7)	2623	(14.3)
1985–89	5028	(16.4)	3000	(16.3)
1990–94	5549	(18.1)	3434	(18.7)
1995–99	5808	(18.9)	3865	(21.0)
2000–02	2721^7^	(8.9)	1983^8^	(10.8)
Not known	959	(3.1)	-	

^1 ^Where date of birth given ^2 ^Includes 486 women whose first pregnancy was conceived after 31^st ^March 2000, 126 of whom were currently pregnant for the first time at time of survey ^3 ^Includes 329 women whose first pregnancy was conceived after 31^st ^March 2000, 73 of whom were currently pregnant for the first time at time of survey ^4 ^Fetal death at <24 weeks gestation. Includes missed miscarriages (fetal death at <24 weeks without spontaneous expulsion of fetus) and blighted ova (anembryonic pregnancy) ^5 ^Termination of pregnancy because of a defect identified in the baby, or because continuing the pregnancy would put the mother's health at risk ^6 ^Termination of pregnancy for reasons other than a defect identified in the baby or risk to mother's health ^7 ^1,718 of these pregnancies were conceived after 31^st ^March 2000 ^8 ^1,232 of these pregnancies were conceived after 31^st ^March 2000

### Stage 2

11,424 (88%) women ever attempting to have children (successfully or unsuccessfully) agreed to participate in the second stage of the study. Of these 596 (5%) were not sent a Stage 2 questionnaire, 212 because they had only ever had one or more termination of pregnancy for non-medical reasons, and 384 because their Stage 1 form arrived back after mailing had ended. A total of 10,828 women were thus sent a second stage questionnaire. The response to this second stage was high (73%), though 2% of women had decided that they no longer wished to participate (Table 1). The 7,702 women completing a Stage 2 questionnaire, and the 18,391 pregnancies they reported, are described in Table 2. Their characteristics are almost identical to those of Stage 1, indicating that Stage 2 responders were an apparently unbiased subset of those responding to Stage 1. 5,777 (75%) women responding to Stage 2 gave signed consent for us to access their medical notes, with 6,963 (90%) agreeing to be contacted again in the future, if required.

### Comparison with national data

Comparisons of Stage 1 data, and the subset Stage 2 data, with national rates are presented in Table 3. There was no evidence to suggest that stillbirth differed from expectation in either Stage 1 (SRSR 115 (95% CI 94 – 139), P = 0.17), or Stage 2 data (SRSR 102 (95% 79 – 132), P = 0.86). Multiple delivery was also in line with expectation from national rates for both stages (Stage 1 SMDR 111 (95% CI 99 – 126), P = 0.08), Stage 2 SMDR 108(95% CI 93–126, P = 32)). Although the inference from this is unambiguous for both stages of the study, the point estimates were noted to be closer to unity for Stage 2 data where almost all pregnancies had known gestational age. This reflects the fact that there might be some slight misclassification of registered stillbirth prior to 1993 in the Stage 1 data where gestational age was only known for 61% of reported stillbirths, some of which might legally be classified as miscarriages.

**Table 3:** Comparison with population birth data of reported births in Stages 1 and 2^1 ^of the National Women's Health Study occurring since 1980^2^

**REGISTERED STILLBIRTH^3^**					
		No. stillbirths^3^	Total livebirths & stillbirths^3^	SRSR^4 ^(95% CI)	

*Stage 1*	1980–2002	102	18,740	115	(94 – 139)
*Stage 2*	1980–2002	59	12,061	102	(79 – 132)

**MULTIPLE (REGISTERED) DELIVERY^5^**					

		No. multiple deliveries^5^	Total deliveries^5^	SMDR^4 ^(95% CI)	

*Stage 1*	1980–2002	264	18,391	111	(99 – 126)
*Stage 2*	1980–2002	169	11,887	108	(93 – 126)

**AVERAGE MATERNAL AGE AT FIRST^6^(LIVE)BIRTH (years)**					

		No. first^6 ^livebirths	Mean (SD) age^7^	England & Wales^8 ^ Mean age	

*Stage 1*	Year of delivery				
	1980–84	1,724	25.2 (4.12)	25.5	
	1985–89	1,916	25.9 (4.56)	26.4	
	1990–94	2,058	27.1 (4.85)	27.8	
	1995–99	2,026	28.6 (5.01)	29.0	
	2000–02	699	29.4 (5.06)	29.6	
*Stage 2*	1980–84	1,032	25.5 (4.02)	25.5	
	1985–89	1,182	26.0 (4.45)	26.4	
	1990–94	1,325	27.3 (4.78)	27.8	
	1995–99	1,432	28.8 (4.81)	29.0	
	2000–02	540	29.7 (4.89)	29.6	

^1^ Stage 2 data are a subset of Stage 1 data (see methods). ^2^ Pregnancies with missing maternal age have been excluded from this analysis. ^3^ Registered stillbirths 1980–2002, defined as fetal death at ≥ 28 weeks prior to 1992, or at ≥24 weeks thereafter. 41 (40%) of stillbirths had no gestational age, but were described as stillbirths by the mother. Unit of analysis is a baby; multiple births counted as many times as there are babies. Denominator contains all reported livebirths and registered stillbirths 1980–2002. ^4^ Standardised Registered Stillbirth Ratio (SRSR) and Standardised registered Multiple Delivery Ratio (SMDR). Standardised for maternal age (5-year intervals) and single year of birth using data for England and Wales 1980–2002. ^5 ^Unit of analysis is a delivery (pregnancy) containing one or more registered live or stillbirth; multiple pregnancies counted once only. Multiple pregnancies containing only one registered birth (with another non-registrable outcome, such as miscarriage) considered as singleton in this analysis. ^6^ First registered birth, if live. ^7^ NWHS data relates to livebirths both within and outside marriage ^8 ^Livebirths within marriage only

Age at first (live) birth was remarkably similar to national data for both Stage 1 and Stage 2 data (Table 3). Exactly as expected, though showing no evidence to suggest any biases with respect to maternal age, average age at first birth was very slightly higher for the national data, since it related to births within marriage only, whereas the NWHS data related to all births (marital status at delivery was unknown).

## Discussion

Using a novel method, the National Women's Health Study has enabled a large UK population-based dataset to be assembled, comprising full reproductive histories, including any history of infertility, for 13,035 women, 12,695 of whom had conceived 30,661 pregnancies. We have obtained further detailed information for 7,702 of these women (18,391 pregnancies), including fertility diagnoses for both male and female partner (if appropriate), and lifestyle and behavioural risk factors for the most recent pregnancy. Seventy-five percent of these women consented to their medical notes being accessed in relation to information reported in the questionnaire, and 90% agreed to be contacted again, thus providing the means to carry out a population-based cohort study of these women at some time in the future.

UK population-based data, collected at government level by England & Wales, Scotland and Northern Ireland, relate to registered births (live and still) and terminations of pregnancy, with Scotland also routinely collecting maternity data on hospital deliveries at any gestation. The National Women's Health Study goes one step further than this, providing the whole reproductive picture. Rather than being a pregnancy-based, cross-sectional survey, the data collected for each woman covers the complete spectrum of reproductive outcomes from infertility problems through miscarriage, ectopic pregnancies and terminations (for both medical and non-medical reasons), to live and stillbirths, and does not rely on legal definitions for inclusion in the dataset. Furthermore, unlike most epidemiological studies of adverse reproductive outcome such as miscarriage, the data source is not clinical (which, for miscarriage, leads to inevitable biases relating to gestational age), but relates to women selected randomly from the UK electoral register. And for outcomes such as infertility no other data currently exist to enable estimation of how many pregnancies in the population as a whole result from fertility treatment.

The study does rely on maternal recall and this could be a source of bias. Studies of self-reported reproductive history and exposures relating to reproductive events have, however, found maternal recall to have acceptably high reliability, and to be little affected by time from event [24-26].

In terms of the key reproductive indicators of stillbirth, multiple delivery rates and maternal age at first birth, the data look remarkably similar to the general population. We therefore feel confident that response was unlikely to be related to adverse reproductive outcome. Indeed, the average age at survey of around 40 years, coupled with average ages at first birth which are exactly as would be expected from general population data, could be seen to indicate that non-responders to the survey tended to concentrate among younger women who had not yet tested their fertility. In addition, we feel confident that those responding to the more detailed Stage 2 questionnaire are an unbiased sample of those responding to Stage 1. Both Stage 1 and Stage 2 data can thus can be considered unbiased with respect to reproduction, and representative of patterns among all women in the UK population who have ever tried to have children, hence prevalence estimates might be taken as unbiased estimates of hard-to-measure outcomes such as miscarriage and pregnancies conceived through assisted reproduction techniques. Such data will be invaluable as population-based reference data for epidemiological studies of reproduction.

In addition to both pregnancy-and woman-based population prevalence estimates, further papers to follow include reports of case-control analyses of behavioural and lifestyle risk factors for miscarriage.

## Conclusions

In summary, we have assembled a large population-based dataset of women's reproductive histories which appears representative of the general UK population and which will enable investigation of hard-to-measure outcomes such as miscarriage and infertility.

## Competing interests

None declared

## Authors' contributions

NM and PD initiated the research and participated in protocol design, data collection, analysis and writing the paper. SP participated in data collection and analysis. All authors read and approved the final manuscript.

## Pre-publication history

The pre-publication history for this paper can be accessed here:


